# Supplementary material for: Identifying adolescents’ gaming preferences for a tobacco prevention social game: A qualitative study
Source: PLoS One. 2023 Jul 28;18(7):e0289319. doi: 10.1371/journal.pone.0289319 (PMC10381079; doi:10.1371/journal.pone.0289319)
Supplement: S1 Table — This file presents the table of consolidated criteria for reporting qualitative research (COREQ). (DOCX) [file pone.0289319.s001.docx]

**Supplementary File 1:** COREQ checklist

| **Items** | **Information** | **Location in manuscript** |
| --- | --- | --- |
| **Domain 1: Research team and reflexivity** |  |  |
| **Personal Characteristics** |  |  |
| 1. Interviewer/facilitator: Which author/s conducted the interview or focus group? | For Phase 1, one lead researcher (GEK, gender: male, credentials: M.P.H., Ph.D.) and one research assistant (JK, gender: female, credentials: B.S.) conducted the sessions. For Phase 2, the lead researcher (GEK, gender: male, credentials: M.P.H., Ph.D.), a graduate research assistant (DM, gender: male, credentials: M.A.), and a research coordinator (ER, gender: female, credentials: B.S.) conducted the sessions. | Materials and Methods, Data Collection, page 9, line 184 |
| 2. Credentials:  What were the researcher’s credentials? E.g. PhD, MD | The researchers on this project had the following credentials: B.S., M.A., M.P.H., and Ph.D. | Title page, page 1 and Materials and Methods, Data Collection, page 9, line 184. |
| 3. Occupation: What was their occupation at the time of the study? | Research Assistant, Research Coordinator, and Assistant Professor. | Materials and Methods, Data Collection, page 9, line 184 |
| 4. Gender: Was the researcher male or female? | In Phase 1, the main interviewer was male, and the secondary interviewer was female. In Phase 2, the main interviewer was female, and the secondary interviewer was male. | Materials and Methods, Data Collection, page 9, line 184 |
| 5. Experience and training: What experience or training did the researcher have? | At the time of the interviews, researchers had full training in the principles of qualitative research. | Materials and Methods, Data Collection, page 9, line 185 |
| **Relationship with participants** |  |  |
| 6. Relationship established: Was a relationship established prior to study commencement? | Yes. A relationship was established through ice-breaker activities prior to the beginning of the study. | Materials and Methods, Data Collection, page 9, lines 190-193 |
| 7. Participant knowledge of the interviewer: What did the participants know about the researcher? e.g. personal goals, reasons for doing the research | Participants were briefed on the purpose of the study and understood that it was a research project about tobacco prevention and that their feedback would be used in the development of a board game. | Materials and Methods, Data Collection, page 9, lines 195 |
| 8. Interviewer characteristics: What characteristics were reported about the interviewer/facilitator? e.g. Bias, assumptions, reasons and interests in the research | There were no interviewer-related biases identified in this study. | Materials and Methods, Data Collection, page 9, lines 187 |
| **Domain 2: study design** |  |  |
| 9. Methodological orientation and Theory: What methodological orientation was stated to underpin the study? e.g. grounded theory, discourse analysis, ethnography, phenomenology, content analysis | In this study, a codebook was first developed based on expectations from the extended-elaboration likelihood model (E-ELM) and the health belief model (HBM). Then, open coding with thematic content analysis was conducted in order to identify new themes. Following grounded theory, as new themes emerged, the codebook continued to evolve, as part of an iterative, inductive process. This process continued until thematic saturation was reached. Supportive of the grounded theory, retrieved themes are deemed appropriate as they provide new concepts that extend E-ELM and HBM. | Materials and Methods, Qualitative Analysis, page 10, lines 208-216 |
| **Participant selection** |  |  |
| 10. Sampling: How were participants selected? e.g. purposive, convenience, consecutive, snowball | In Phase 1, participants were recruited from a youth organization in Houston, Texas. In Phase 2, participants were recruited from youth organizations in Florida, as well as a registry of potential research participants from underserved Florida counties. | Materials and Methods, Recruitment and Sampling Strategy, page 7, line 150 |
| 11. Method of approach: How were participants approached? e.g. face-to-face, telephone, mail, email | In Phase 1, participants were approached face-to-face. In Phase 2, as a result of the study being conducted during the COVID-19 quarantine, participants were approached through email and phone contact. | Materials and Methods, Recruitment and Sampling Strategy, page 7, line 152 |
| 12. Sample size: How many participants were in the study? | 30 | Results, page 11, line 233 |
| 13. Non-participation: How many people refused to participate or dropped out? Reasons? | Two participants dropped out during the phase 2 sessions because they were no longer interested in the content of the session. One participant in Phase 2 refused to complete the survey, but did participate in the session. | Materials and Methods section, under Data Collection, page 9, lines 190-194 |

| **Setting** |  |  |
| --- | --- | --- |
| 14. Setting of data collection: Where was the data collected? e.g. home, clinic, workplace | In Phase 1, sessions were conducted in-person at a youth organization, in a private room. In Phase 2, sessions were conducted online using the video conferencing software Zoom. Quantitative data was collected using an online survey. Participants engaged in the study in a private room from home. | Materials and Methods section, under Data Collection, page 9, lines 196 |
| 15. Presence of non-participants: Was anyone else present besides the participants and researchers? | No | Materials and Methods section, under Data Collection, page 9, lines 196 |
| 16. Description of sample: What are the important characteristics of the sample? e.g. demographic data, date | Phase 1 participants were 73.33% male with a mean age of 12.93 years (SD = 0.80). All participants in Phase 1 identified as African-American or Black. In Phase 2, participants were 57.14% female, and they had an average age of 14.71 years (SD = 2.33). | Results, page 11, line 234 |
| **Data collection** |  |  |
| 17. Interview guide: Were questions, prompts, guides provided by the authors? Was it pilot tested? | For Phase 1, a semi-structured instrument was pilot-tested with two young-adult volunteers and 5 adolescents, and then revised for completion, with 22 questions.  For Phase 2, a qualitative instrument was created and pilot-tested with two young-adult volunteers and two adolescents, and then revised for completion, with 35 questions. | Materials and Methods, Interview Instrument, page 8, line 176 |
| 18. Repeat interviews: Were repeat interviews carried out? If yes, how many? | No. | Materials and Methods, Data Collection, page 10, line 205 |
| 19. Audio/visual recording: Did the research use audio or visual recording to collect the data? | In Phase 1, the focus group discussions were audio recorded using a handheld audio-recorder. In Phase 2, the interviews were audio and video recorded using the native record feature on Zoom. A backup recording was made using a third-party software. | Materials and Methods, Data Collection, page 9, line 189 |
| 20. Field notes: Were field notes made during and/or after the interview or focus group? | Yes, additional field notes were made. | Materials and Methods, Data Collection, page 10, line 204 |
| 21. Duration: What was the duration of the interviews or focus group? | The qualitative sessions lasted approximately 2 hours. | Materials and Methods, Data Collection, page 10, line 203 |
| 22. Data saturation: Was data saturation discussed? | Yes, coding continued until thematic saturation was reached. | Materials and Methods, Qualitative Analysis, page 10, line 223 |
| 23. Transcripts returned: Were transcripts returned to participants for comment and/or correction? | No. | Materials and Methods, Qualitative Analysis, page 11, line 230 |
| **Domain 3: analysis and findings** |  |  |
| **Data analysis** |  |  |
| 24. Number of data coders: How many data coders coded the data? | Two coders coded for Phase 1. The two coders in Phase 1 coded for Phase 2 with an additional new coder, which led to 3 coders for Phase 2. | Materials and Methods, Qualitative Analysis, page 10, line 211 |
| 25. Description of the coding tree: Did authors provide a description of the coding tree? | Open coding with thematic analysis and the grounded theory approach. Coding was described in the Methods section, and a codebook is provided as a supplementary file. | S2 File and Materials and Methods, Qualitative Analysis, page 10, line 208-230 |
| 26. Derivation of themes: Were themes identified in advance or derived from the data? | Some themes were derived in advance, and then additional themes were derived from the data. | Materials and Methods, Qualitative Analysis, page 10, line 208-230 |
| 27. Software: What software, if applicable, was used to manage the data? | Microsoft Word, Microsoft Excel, and Nvivo. | Materials and Methods, Qualitative Analysis, page 11, line 230 |
| 28. Participant checking: Did participants provide feedback on the findings? | Infographic materials were sent to participants after data analysis. Feedback on these findings has not yet been received. | Materials and Methods, Qualitative Analysis, page 11, line 229 |
| **Reporting** |  |  |
| 29. Quotations presented: Were participant quotations presented to illustrate the themes / findings? Was each quotation identified? e.g. participant number | Yes, themes were supported with direct quotes attributed to anonymized participants by gender and focus group number. | Results, page 13-19, lines 250-392 |
| 30. Data and findings consistent: Was there consistency between the data presented and the findings? | Yes | Results, page 13-19, lines 250-392 |
| 31. Clarity of major themes: Were major themes clearly presented in the findings? | Yes, major themes were presented in the Results section. | Results, page 13-19, lines 250-392 |
| 32. Clarity of minor themes: Is there a description of diverse cases or discussion of minor themes? | Minor themes are discussed in the manuscripts. | Results, page 13-19, lines 250-392 |
