# Supplementary material for: Identifying adolescents’ gaming preferences for a tobacco prevention social game: A qualitative study
Source: PLoS One. 2023 Jul 28;18(7):e0289319. doi: 10.1371/journal.pone.0289319 (PMC10381079; doi:10.1371/journal.pone.0289319)
Supplement: S1 File — This file presents the qualitative semi-structured instruments for the study. (DOCX) [file pone.0289319.s002.docx]

**Qualitative Semi-structured Instruments**

### Phase 1

### INTRODUCTION OF SESSION (1 minute)

***After going through the consent document in detail, the moderator or interviewer will make an introduction:*** I want to thank you for taking the time to meet with me today. So together today, we want to get your opinion about games and tobacco.

Our discussion should not take longer than an hour. I will be recording the session because I don’t want to miss any of your comments.

Although I will be taking some notes during the session, I can’t possibly write fast enough to get it all down. Because we’re recording, please be sure to speak up so that we don’t miss your comments. Please avoid mentioning anyone’s name. All responses will be kept confidential. This means that your answers will only be shared with the research team members and we will make sure that any information we include does not identify you as the respondent. Remember, you don’t have to talk about anything you don’t want to and you may end the interview at any time. Do you have any questions before we start?

### WARM-UP QUESTIONS (3 minutes)

*You will warm them up with some general questions. Give about 1 minute to each bullet point.*

- What are some activities that you like to participate in? This could be at school, at home, at the summer camp, or even on your phone or computer.
- What are some activities that you like to do with friends?

**HOW THEY PLAY GAMES AND THEIR FEATURES (5 minutes)**

*Give about 1 minute for every bullet point.*

- Where and when do you play games? What type of games do you play?
- What are some games that you like to play?
- Are there any games that you like to play with friends? Are there any games that you like to play alone?
- What do you like the most about games? Why would you play a game again and again? What do they have in them that makes you enjoy them?

**HOW THEY PLAY BOARD GAMES AND THEIR FEATURES (5 minutes)**

*Give about 1 minute for every bullet point.*

- Have you ever played a board game, like Monopoly, Uno, Chess, Card games, Battleship, Life…?
- Which board games have you played before?
- Where and when do you play these board games?
- Are there any board games that you like to play with friends? Are there any board games that you like to play alone?
- What do you like the most about board games? Why would you play a board game again and again? What do they have in them that makes you enjoy them?

**PLAYING A BOARD GAME (35 minutes)**

**Play a board game in a group (20 minutes):**

*They will team up in groups and each group would play a board game (if 5 then 1 group of 5, and if 6 then 2 groups of 3)*

- We would like you to first play board games in groups of 3. You have 20 minutes to play the game. After playing, we will talk about these games.

**Feedback on the board game (5 minutes):**

*Twenty minutes later, each moderator will ask questions to each group separately, in parallel.*

- What was this board game about?
- What do you like the most about the board game? Why would you play this board game again and again?
- What are your favorite activities in this game? What did it have in it that made you enjoy it?

**Feedback on our idea (15 minutes):**

*Make sure to get as many answers as possible for each of the following questions:*

- We already have a vague idea for a board game, and we would like your help to advance it. Imagine a board game with a map on it, and your mission is to reach the center of the map.
- Where do you imagine you are in this game? Is it a forest, an island, a galaxy, a city, a neighborhood?
- As you move toward the center of the map, you are running away from something bad. What do you think this could be?
- You want to reach the center of the map to escape. What could be in the center of the map that can help you escape? What else?
- What character of a story would you imagine you are in this game?
- On your journey toward the center, what are some challenges that you imagine facing as you move forward?
- You will not be competing against your group members in this game. Instead, you have to help them reach the center with you. You will be playing together, against the game. What do you think of this idea?
- How could you help your group members face the challenges you just mentioned?

**Phase 2**

**Introduction**

1. **Disclaimers**
   1. “Hi everyone! Thank you for joining us today for this group discussion! Before we start, I would like to go over a few important points.”
   2. As we engage in this group discussion, please remember that all opinions are very valuable to us; there are no right or wrong answers, we just want to learn about your thoughts, so please don’t be afraid to speak up! Although, you are not required to answer questions that make you uncomfortable.
   3. Everything you say or type in Zoom is confidential. Your parents, teachers, or others at your program will not know what was said in this meeting. Only research staff will have access to this information.
   4. Do you have any questions so far?
2. **Intro to topics discussed in focus group**
   1. In this group discussion, you will answer questions and participate in activities to help us design a new board game! During the activities, we will ask you to draw, so please have a piece of paper and a pencil ready.
   2. Do you have any questions so far?
3. **Brief overview of Zoom**
   1. What to do if you get disconnected from Zoom
   2. Do you have any questions so far?
4. **Introduction of members in focus group**
   1. To get to know each other better, let's introduce ourselves by saying your name and one fun fact about yourself. **For example, interests, hobbies, favorite things…**

**Main Questions**

**Board game design/content**

*Provide an overview of storyline, rulebook, characters, board game- Only go over each section at a time*

1. Storyline:

a. What are your thoughts/feelings about the story so far?

b. In this story, what do you think the island will look like?

c. So this was an introduction to a board game. Can you tell me how you imagine this board game to be?

i. What do you think you will be doing on the island to protect it from the storm?

2. Island

a. Let’s take a quick look at the island.

i. What do you think of the island?

ii. What would you want to change in this island to make it better?

1. Is there anything missing from this island that you think should be there?

3. Characters

a. What do you think of the characters? *Ask this question in general for all characters*

b. If you could change anything about these characters, what would it be?

c. If you could add in a new character that you think would fit the story, can you describe them to us?

i. What would the character look like?

ii. What accessories would they have?

4. Rulebook:

a. Instruction: I will go over the rules of the game. Please listen carefully. IIf there are rules that you do not understand, please feel free to ask questions after the section that I have discussed.

b. Questions for each section

i. Does anyone have any questions so far?

c. General questions, *after going through the entire rulebook:*

i. What do you all think of this game?

ii. How easy is it to understand the rules of the game?

iii. What do you feel is complicated in this game?

1. What can be done to make it less complicated?

5. Trivia mini-game

a. Interactive-Activity: Play-test the Storm and Knowledge Cards

i. For this activity, I will be sharing with you a series of mini-game cards that have information on them. Then, I will share my screen to show you a trivia question. You all will be working together as a team to solve this trivia question. You will have one minute to find the answer to the question by using your mini-game cards.

ii. After the game, we will have a discussion about:

1. The gameplay/mini-game cards information

a. What did you think about the information on the mini-game cards?

i. How easy was it to solve the trivia question using the information?

ii. How easy was it to understand the information on any of these mini-game cards?

1. What would you want to change on the mini-game cards to make them better?

b. How can we make this game more fun?

2. Mini-game cards design

a. What did you think about the mini-game cards design?

b. What are some ways you would change the design of the mini-game cards to better suit the game?

b. Interactive-Activity: Mini-Game Cards

For this activity, I will send four of you a mini-game card and we will send it one at a time. You will have 45 seconds to play the game. *If they cannot get it in 45 seconds, give them additional time without letting them know we went past 45 seconds.*

1. There are 4 types of games you could get:
   1. The first game is called “Acting”: Without speaking, try to act out the word on your mini-game card.
   2. The second game is called “drawing”: Without speaking, try to draw something that would guide the other participants to guess what the word on your mini-game card is.
   3. The third game is called “speak out”: Try to describe the word(s) given on your mini-game card to the best of your abilities without using the words next to the text that says “DON’T USE:”.
   4. The fourth game is about friendship.
   5. Do you have any questions? Are you ready?

1. Raise your hand if you have been sent the mini-game. *Call on participant #1 to play the “acting” game.* You will have 1 minute to play the game. I will tell you when it is time to start.
   1. What did you think about this game?
      1. What about the mini-game was most difficult to play through?
      2. What are some things about the mini-game that can be improved?
      3. For those who weren't acting, what would you change about this game to make guessing the word more fun?

1. Raise your hand if you have been sent the mini-game. *Call on participant #2 to play the “drawing” game.* You will have 1 minute to play the game. I will tell you when it is time to start.
   1. *If they win this mini-game tell them “congratulations! You can add 1 heart to the island. You are one step closer to healing the island”*

*If they lose this mini-game tell them “tough luck! You will have to add 1 disease to the island.”*

- 1. What did you think about this game?
     1. What about the mini-game was most difficult to play through?
     2. What are some things about the mini-game that can be improved?
     3. For those who weren't drawing, what would you change about this game to make guessing the word more fun?

1. Raise your hand if you have been sent the mini-game. *Call on participant #3 to play the “speak out” game.* You will have 1 minute to play the game. I will tell you when it is time to start.
   1. *If they win this mini-game tell them “congratulations! You can add 1 heart to the island. You are one step closer to healing the island”*

*If they lose this mini-game tell them “tough luck! You will have to add 1 disease to the island.”*

- 1. What did you think about this game?
     1. What about the mini-game was most difficult to play through?
     2. What are some things about the mini-game that can be improved?
     3. For those who weren't drawing, what would you change about this game to make guessing the word more fun?

III. Raise your hand if you have been sent a mini-game. *Call on participant # 4 to play a friendship game.* You will have 1 minute to play the game. I will tell you when it is time to start.

*A.* *If they win this mini-game tell them “congratulations! You can add 1 heart to the island. You are one step closer to healing the island”*

*If they lose this mini-game tell them “tough luck! You will have to add 1 disease to the island.”*

B. What did you think about this game?

1. What are some things about the mini-game that can be improved?

a) What are some ways to make the mini-game more fun?

2. What about the mini-game was most difficult to play through?

3. For those who weren't saying their 2 truths and a lie, what would you change about this game to make guessing the word more fun?

The mini-game card’s design:

- - 1. What do you like about the design of the mini-game card?
    2. What do you dislike about the design of the mini-game card?
    3. If you were able to improve the design of the mini-game card, what would you do?

C. What do you think the game should be called?
