# Supplementary material for: Identifying adolescents’ gaming preferences for a tobacco prevention social game: A qualitative study
Source: PLoS One. 2023 Jul 28;18(7):e0289319. doi: 10.1371/journal.pone.0289319 (PMC10381079; doi:10.1371/journal.pone.0289319)

Description of the Game Concept

# Storyline

- **Storyline:**
  - You live on a tranquil and calm island in the Pacific Ocean. Your fellow islanders are happy, energetic, and friendly. You live long and healthy lives. Until… one day, a huge storm hits your island! But it wasn’t like any other storm. It brings with it a flurry of tobacco products, carcinogens, smoke, and nicotine. Your friends and neighbors begin to become very sick. Your environment and crops wither. Your entire island is in danger! In order to save the island from the storm’s damage, you and your friends will have to go on quests. Across the island, you will find ways to resist tobacco products and their effects. You’ll have to work together to help your neighbors, fix your environment, and make the island healthy once again. Your island is depending on you!
    - What are your thoughts/feelings about the story so far?
    - In this story, what do you think the island will look like?
    - This was an introduction to a board game. Can you tell me how you imagine this board game to be?
      - What do you think you will be doing on the island to protect it from the storm?
- **Introduction**
- **Set Up the Game**
  - On this island, everyone is **working together as a team**.
  - **Each player chooses a Character Card to represent them.** Your card will let you know where to be on the island.
  - **At the beginning of the game, each of you receives a specific**

**number of random Knowledge Cards** .

- You may show each other these Knowledge Cards at any time.
- Knowledge Cards will become handy when facing trivia questions.
- Start the Game
  - The player whose **birthday is coming up next** starts the game. You can then **take turns moving clockwise**.
  - On your turn, you will **roll the die and move your character forward** that number of spaces on the island.
  - You must then **interact with the space** you land on, depending on the activity on that space.

**There are 3 main spaces on the board for 3 main activities**

1. **
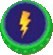
** **Storms:**
   - When you land on a storm space, you will draw a **Storm Card**.
   - A Storm Card will present you with a **trivia question**.
   - The answer to the trivia question is on the card so you are the only one to see it.
   - Your team except yourself must share **Knowledge Cards**

with each other to find the answer to the trivia question.


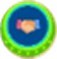
**There are 3 main spaces on the board for 3 main activities**

1. **Teamwork:**
   - When you land on this space, you will draw a **Teamwork Card**.
   - These cards will present you with a development in the story on the island.
   - They will give you and your team a choice - good or bad - that you have to make.
   - Many situations will let you draw additional Knowledge Cards.
   - There is no limit to how many Knowledge Cards can be collected.

**There are 3 main spaces on the board for 3 main activities**

1. **Mini-Games:**
   - When you land on this space, you will draw a Mini-Games Card.
   - Mini-Games can be one of 3 types: drawing, acting, or speaking out.
   - To play a Mini-Game, you must get players on your team to guess the answer listed on the card.
     -
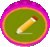

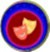
 Drawing: You will need to draw a depiction that allows others to guess the answer on the card.
     - Acting: By miming, you will need to act what is on the card to allow others to guess the answer on the card.
     -
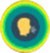
 Speaking out: You will describe what you see on the card without using the same words. Others will have to guess the answer on the card.

**Winning, Losing, and How the Game Ends**

- **There are ten regions on the is land.** Activities in the game will make these places either healthier or sicker.
  - The team wins an activity → the region becomes healthier and receives heart tokens → the team gains number of points = number of heart tokens
  - The team loses an activity → the region becomes sicker and receives disease tokens → the team loses number of points = number of disease tokens
- **How the game ends:** On this island, everyone is working together. Players

will score and lose points as a team. The final score (from 0 to 100) will tell the team how well they succeeded in healing the island.


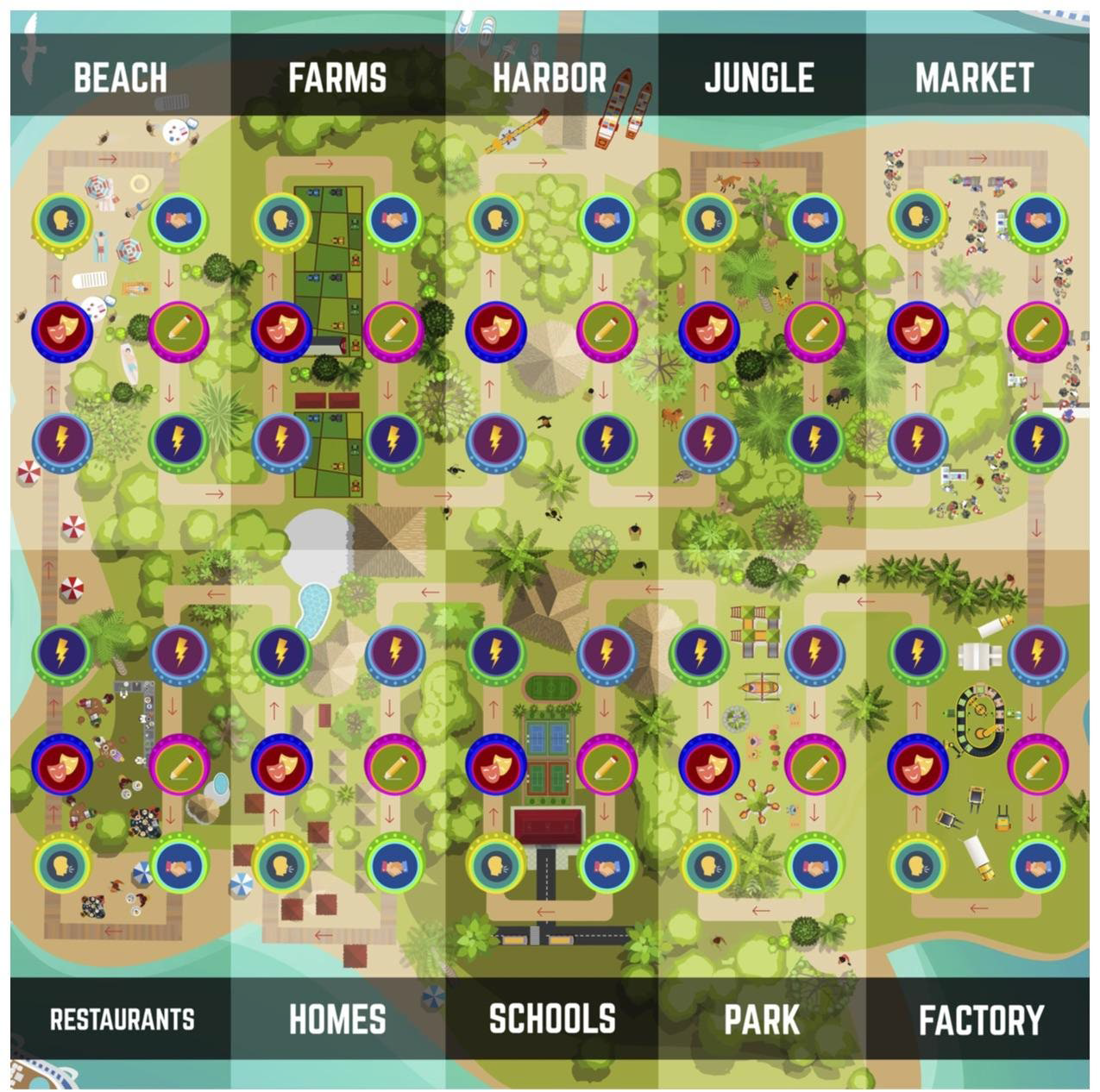


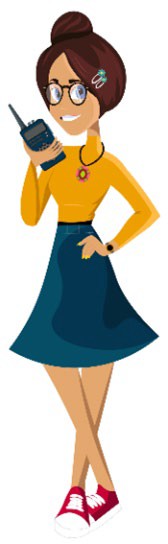

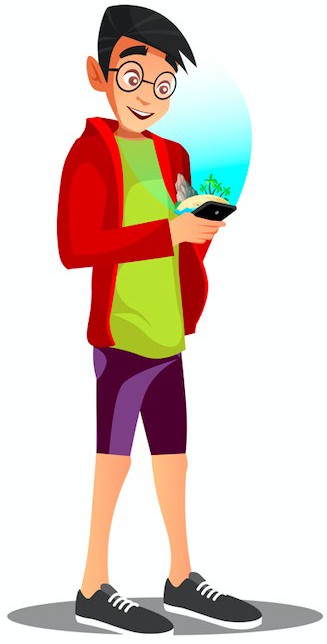

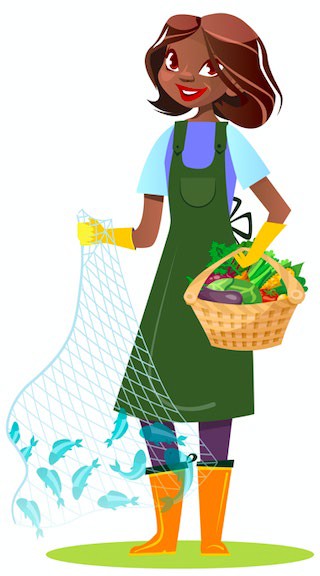

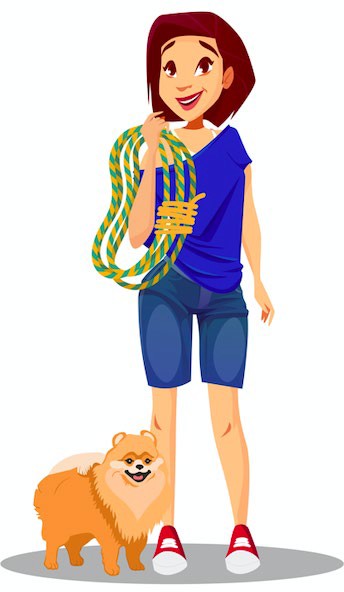


**Group Discussion: Trivia Game**

**Trivia Game**

- Let’s play a trivia game!
- Introduction to the trivia game


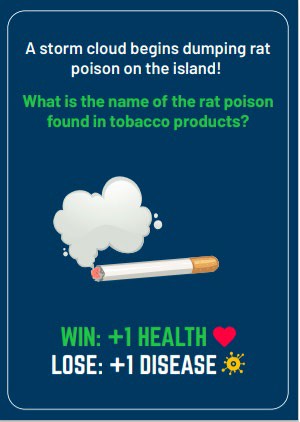


# Trivia Game

- **Trivia Game Questions**
  - What do you think about the information on the trivia question card?
  - How easy was it to solve the trivia question using your knowledge card?
  - What would you change on the trivia question card to make them better?
  - How much fun was this activity?
    - How can we make this game more fun?

# Trivia Card Design


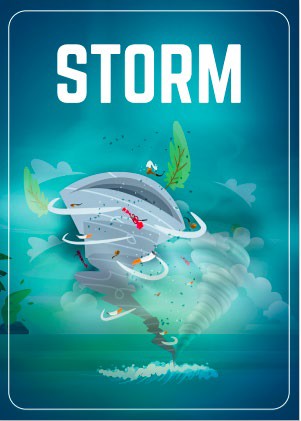

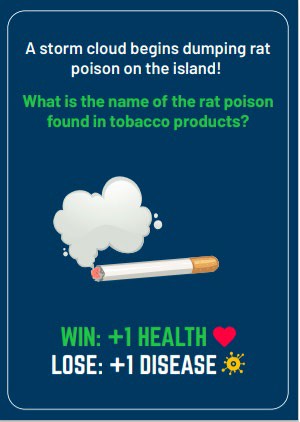

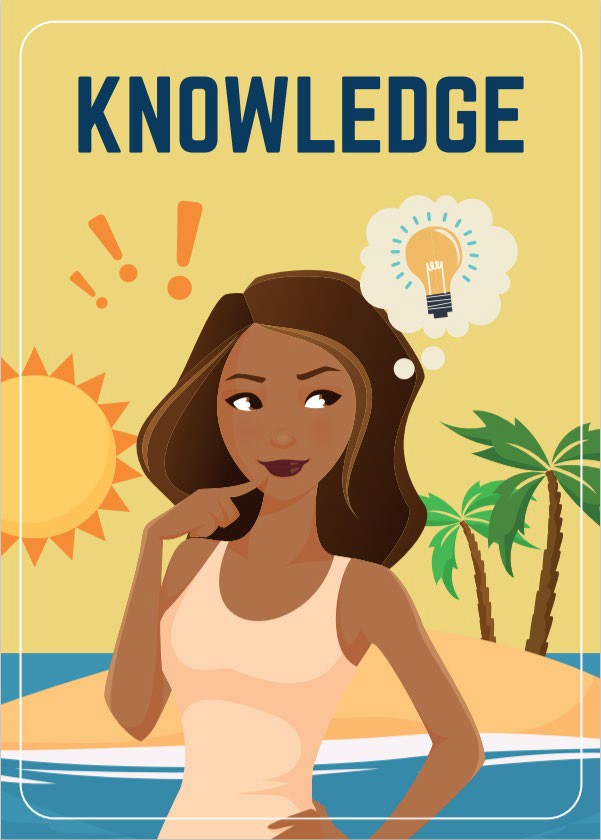

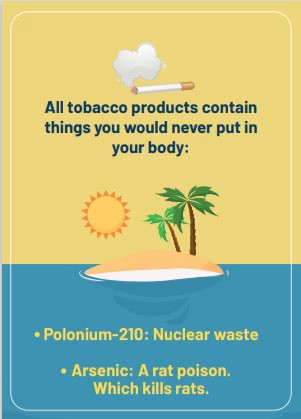


**Mini-Game Cards**

**Mini Games**

There are 4 types of games you could get:

The first game is called “Acting”: Without speaking, try to act out the word on your mini-game card.

The second game is called “drawing”: Without speaking, try to draw something that would guide the other participants to guess what the word on your mini-game card is.

The third game is called “speak out”: Try to describe the word(s) given on your mini-game card to the best of your abilities without using the words next to the text that says “DON’T USE:”.

The fourth game is a special mini game.

#
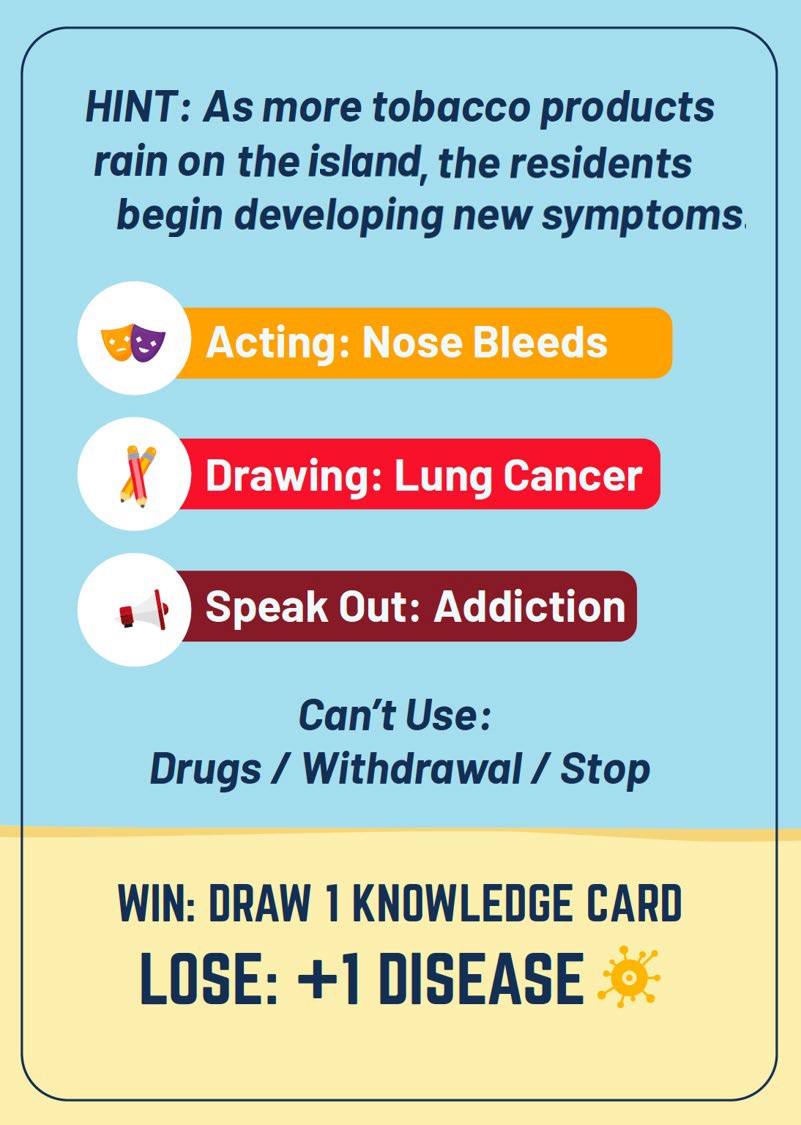
Mini-Game Cards Acting

**Acting:** The first game is called “Acting”: Without speaking, try to act out the word on your mini-game card. You will have 1 minute to play the game. I will tell you when it is time to start.

#
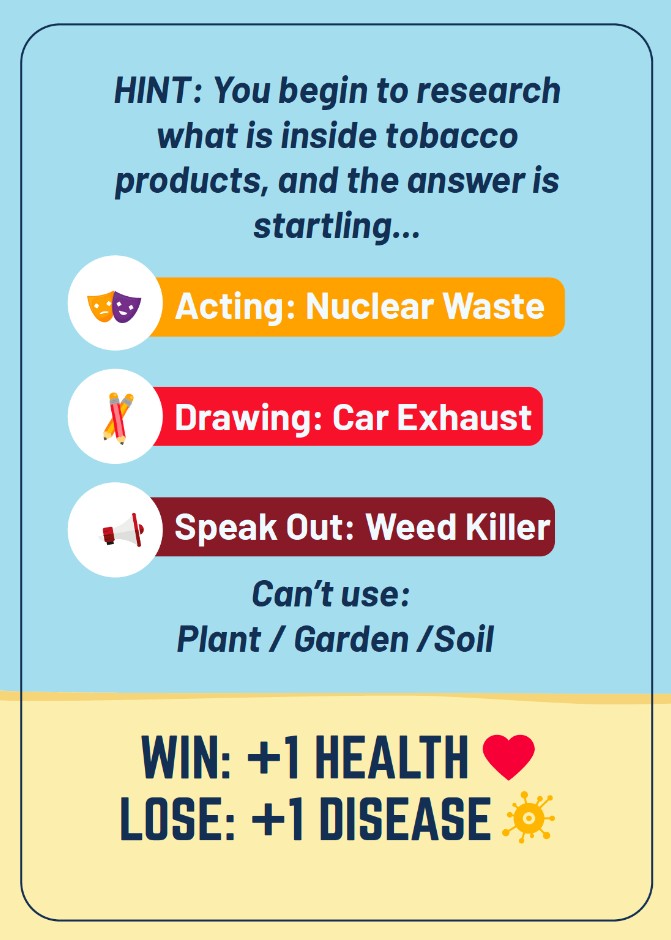
Drawing Mini-Game

- **Drawing:** The second game is called “drawing”: Without speaking, try to draw something that would guide the other participants to guess what the word on your mini-game card is. You will have 1 minute to play the game. I will tell you when it is time to start.

#
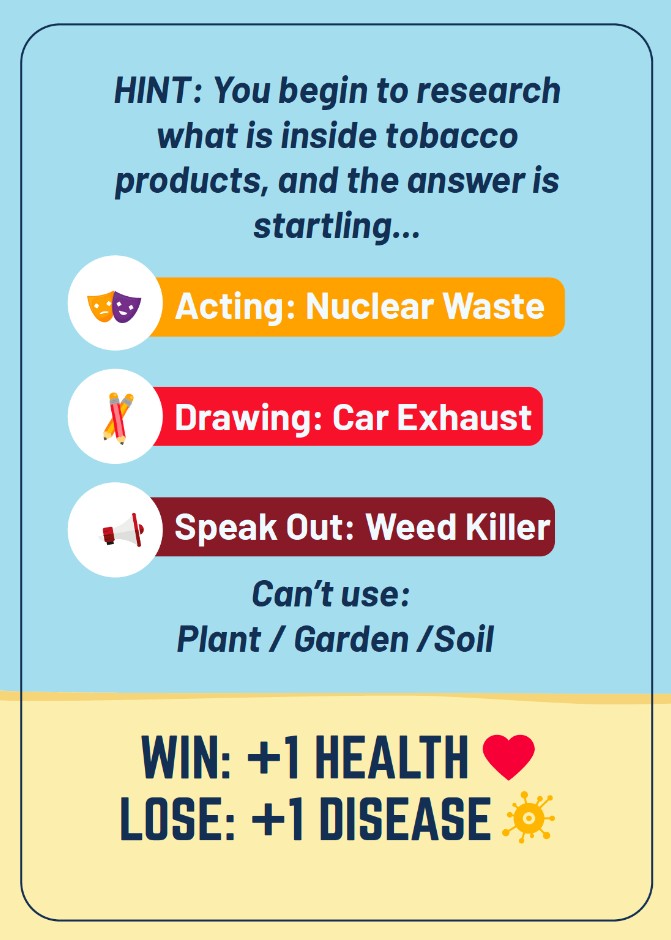
Interactive-Activity: Speak Out Mini-Game

- **Speak out:** The third game is called “speak out”: Try to describe the word(s) given on your mini-game card to the best of your abilities without using the words next to the text that says “DON’T USE”. You will have 1 minute to play the game. I will tell you when it is time to start.

#
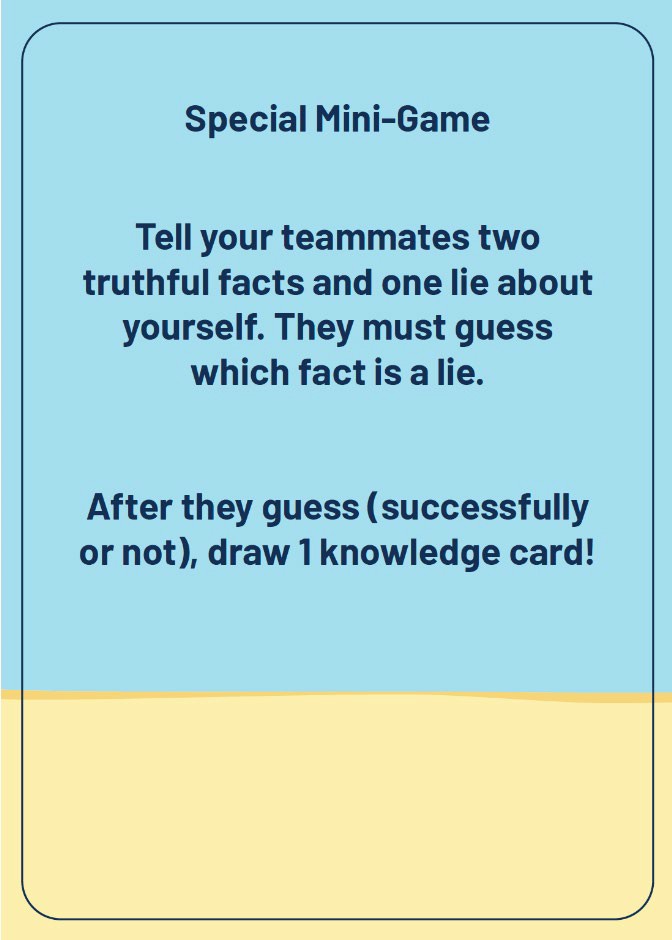
Interactive-Activity: Special Mini-Game

- **Special mini-game:** “Tell your teammates two truthful facts and one lie about yourself. They must guess which fact is a lie. [bottom of card]”

**Card Design**


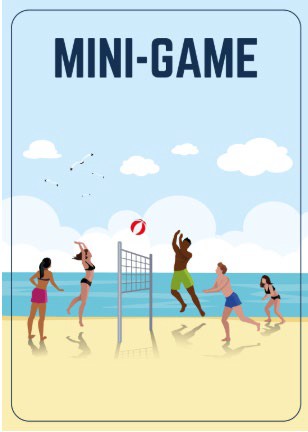

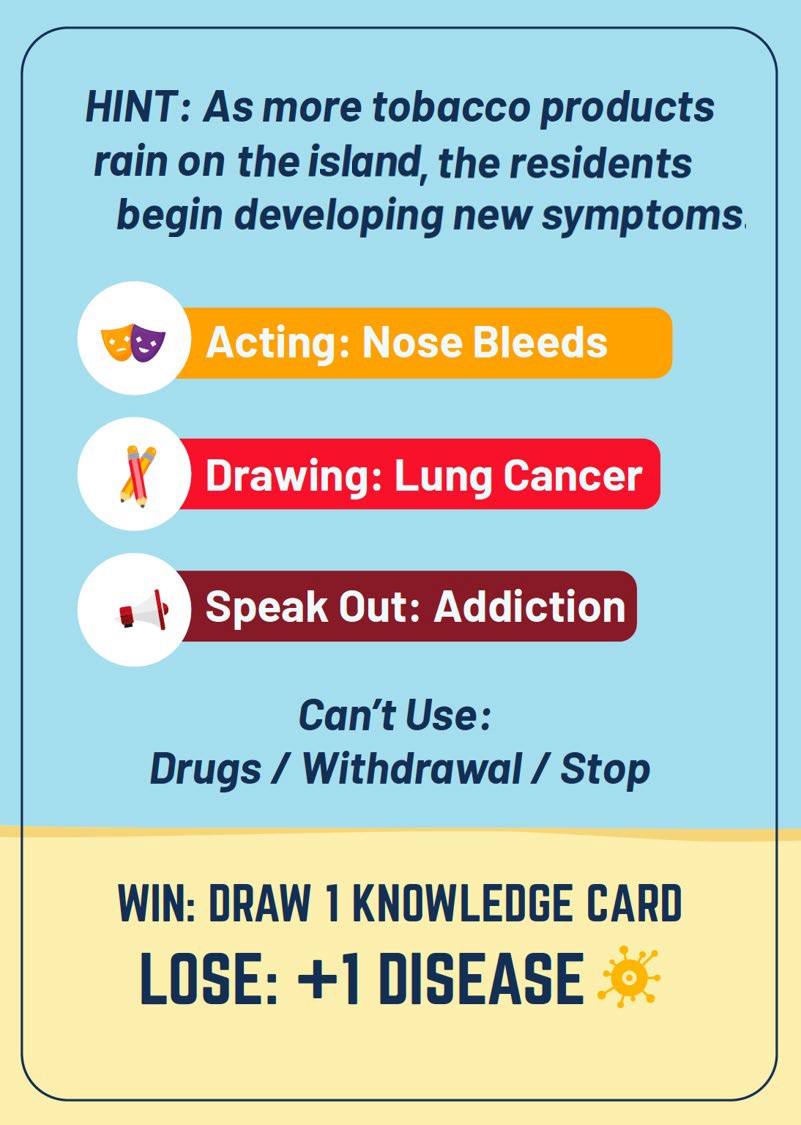

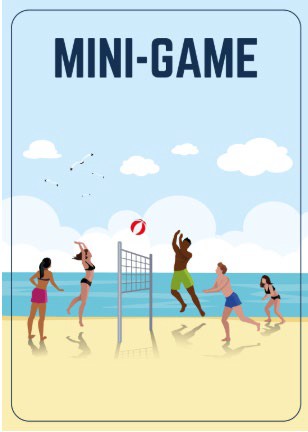

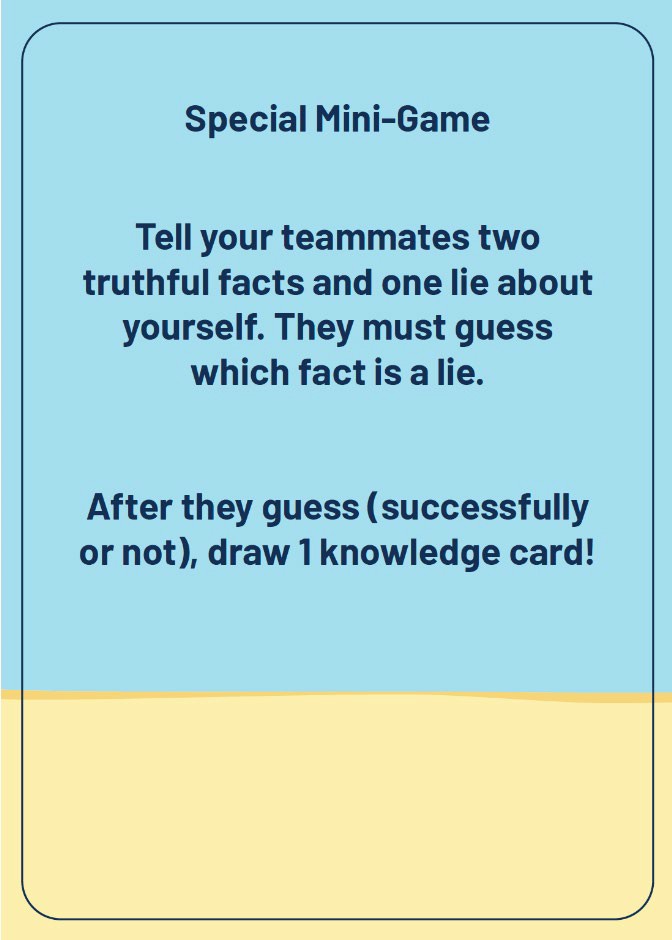

Supplement: S2 File — This file describes the game in detail. (DOCX) [file pone.0289319.s003.docx]
